# Targeting oncogenes in advanced melanoma

**DOI:** 10.1186/1897-4287-10-S2-A24

**Published:** 2012-04-12

**Authors:** GA McArthur

**Affiliations:** 1 Peter MacCallum Cancer Centre, East Melbourne, Australia

## 

The ability to target oncogenes in malignancies such as CML, GIST, APML and ERBB2-positive breast cancer has revolutionized the management of those diseases. Interesting over 70% of melanomas contain genomic amplification or mutations in one of the oncogenes BRAF, NRAS, KIT, CCND1 or CDK4 that may induce an oncogene addicted state. Inhibition of BRAF with Vemurafenib or GSK2118436 in BRAF-mutant melanoma have shown responses in over 50% of patients with advanced disease and in the case of Vemurafenib striking improvements in survival compared to DTIC. These data suggest that there are therapeutically targetable oncogenes in melanoma. However emergence of resistance is common. A number of mechanisms of resistance have been identified including reactivation of the RAS/RAF/MEK/ERK pathway. Current focus is the development of combination strategies including the addition of MEK-inhibitors to BRAF-inhibitors and the combining targeted agents with immunological agents such as Ipilimumab. Together these data indicate that targeting oncogenes in melanoma offers significant therapeutic opportunities in one of the most challenging of human malignancies for systemic therapy.

